# Neurometabolic indicators of mitochondrial dysfunction in repetitive mild traumatic brain injury

**DOI:** 10.2217/cnc-2017-0013

**Published:** 2017-10-04

**Authors:** Susan Kim, Steve C Han, Alexander J Gallan, Jasmeet P Hayes

**Affiliations:** 1 Boston University School of Medicine, Boston, MA 02118, USA; 2 Department of Pathology, University of Chicago Medical Center, Chicago, IL 60637, USA; 3 National Center for PTSD, VA Boston Healthcare System, Jamaica Plain, MA 02130, USA; 4 Department of Psychiatry, Boston University School of Medicine, Boston, MA 02118, USA

**Keywords:** axonal injury, biomarkers, blood–brain barrier, mitochondrial dysfunction, mTBI, rmTBI, TBI

## Abstract

Mild traumatic brain injury (mTBI) is a significant national health concern and there is growing evidence that repetitive mTBI (rmTBI) can cause long-term change in brain structure and function. The mitochondrion has been suggested to be involved in the mechanism of TBI. There are noninvasive methods of determining mitochondrial dysfunction through biomarkers and spectroscopy. Mitochondrial dysfunction has been implicated in a variety of neurological consequences secondary to rmTBI through activation of caspases and calpains. The purpose of this review is to examine the mechanism of mitochondrial dysfunction in rmTBI and its downstream effects on neuronal cell death, axonal injury and blood–brain barrier compromise.

Mild traumatic brain injury (mTBI) is a significant national health concern that accounts for up to 75% of the 1.5 million TBIs sustained in the USA each year, and costs the taxpayers at least US$17 billion annually [1]. Clinical symptoms immediately following trauma include lethargy, disorientation, vertigo, headaches, sensitivity to light and sound, among others [2]. mTBI is diagnosed if post-traumatic amnesia or alteration of consciousness is ≤24 h; if the length of loss of consciousness is ≤30 min; and/or, if a patient scores 13–15 on the Glasgow Coma Scale [3,4].

Most individuals exposed to mTBI recover to baseline within 3 months [5]. However, there is growing awareness that repetitive mTBI (rmTBI), or even repetitive hits to the head without subsequent symptoms of TBI, are associated with chronic effects on the brain and clinical symptoms [6]. Sports injury studies suggest that receiving several concomitant concussions may lead to either cumulative increases or a prolonging of the symptoms seen in single injuries [7–13]. US military personnel serving in Iraq and Afghanistan may be consistently threatened with multiple low-level blast exposure in and around the theaters of war [14,15]. Moreover, recent work in the area of chronic traumatic encephalopathy indicates that repetitive concussions or mTBI may be linked to chronic traumatic encephalopathy [16–18].

Although the acute and long-term behavioral consequences of rmTBI have gained recent attention from researchers, the media and funding agencies, the underlying molecular mechanisms are still largely unknown. The purpose of this review is to synthesize findings relating to mitochondrial dysfunction following rmTBI and downstream effects including neuronal cell death, axonal injury and blood–brain barrier (BBB) compromise. Relevant animal models and human literature will be explored, as well as key methodology used to study aspects of rmTBI and the various mechanisms of injury (Table 1).

**Table 1.** **Summary of studies and their findings in the review regarding mild traumatic brain injury, repetitive mild traumatic brain injury and mitochondrial dysfunction.**

**Study (year)**	**Type of injury**	**Subjects information**	**Method of injury**	**Study findings**	**Ref.**
Vagnozzi *et al*. (2008)	mTBI and rmTBI, traumatically induced alteration in mental status, with clinical symptoms of concussion, with post injury MRI negative for intracranial lesion	11 mTBI human subjects athletes, ages 21–35; 3 rmTBI human subjects, ages 25–30	Sports-related concussion	NAA/Cr ratio in singly concussed athletes was lower compared with control subjects at 3 days postinjury and recovered to control values at 30 days postinjury; second injury (which occurred at 10, 12 and 13 days from the first one) was associated with a further decrease in NAA/Cr levels post second injury with full restoration taking place at 45 days post initial injury	[12]

Vagnozzi *et al*. (2007)	rmTBI, drop-weight (450 g at 1-m height) without postinjury skull fracture, seizure, nasal bleeding or death	42 Wistar male rat subjects weighing 300–350 g, ages not stated	Drop-weight	ATP, NAA and acetyl-CoA levels were maximally decreased when the interinjury interval was 3 days, even after a 7-day recovery period post second injury; by contrast, rats subjected to an interinjury interval of 5 days did not show differential levels compared with the control group	[23]

Prins *et al*. (2013)	mTBI and rmTBI, controlled pneumatic piston cylinder injury 36 psi without skull fracture	33 Sprague–Dawley rat subjects weighing 142.9 ± 3.9 g, age 35 days	Controlled pneumatic piston cylinder	Rats subjected to a single mTBI show decreased CMRgluc in the parietal cortex and the hippocampus; CMRgluc levels decreased further when rats sustain a second injury within 24 h of the first injury, and remain decreased 3 days postinjury whereas rats subjected to a single mTBI returned to normal CMRgluc values; in contrast, rats that were subjected to 120-h interval rmTBI showed no significant changes compared with that in the group with single mTBI; the CMRgluc in this rmTBI group returned to the levels seen in the uninjured group 3 days after the second injury	[24]

Peskind *et al*. (2011)	rmTBI, at least one blast exposure history that resulted in acute mTBI according to the ACRM critera except the GCS requirement	12 human subjects Iraq war veterans, age 32 ± 8.5 years	Blast injury	Subjects with blast-induced rmTBI exhibited decreased CMRgluc compared with control subjects with no history of brain injury; the decrease in CMRgluc appears to be long-lasting as the average time between the veterans' last blast exposure and the time at which CMRgluc was measured was 3.5 years	[25]

Govindaraju *et al*. (2004)	mTBI, injury with GCS 13–15 and LOC less than 30 min, none with post-traumatic amnesia more than a few hours	14 human subjects, ages 18–53 years	Various (MVA, fall, assault)	Decreases in NAA/Cr and NAA/Cho, markers of neuronal damage or disruption, in 14 human subjects with mTBI compared with 13 control subjects when measured within 1 month postinjury	[32]

Son *et al*. (2000)	mTBI, regional brain contusion with initial GCS 13–15	7 human subjects, ages 15–60 years	Various (MVA, fall, assault)	Reduction in NAA/Cr ratios in pericontusional regions in 7 mTBI human subjects compared with 25 control subjects	[33]

Lazzarino *et al*. (2012)	rmTBI, sport-related concussion inducing a transient alteration in mental status, GCS 14–15 and normal neurological objective signs	6 human subjects atheletes, ages 20–33 years	Sports-related concussion	Repeat concussion increased the time of recovery of NAA/Cr to baseline as well as the persistence of clinical symptoms	[34]

Hinzman *et al*. (2010)	mTBI, 1.1 ± 0.01 atm; moderate TBI 2.0 ± 0.02 atm	21 male Sprague–Dawley rats, weighing 350–400 g, ages not stated	Fluid-percussion injury	Mild brain injury significantly increased tonic glutamate levels and the levels correlate with the injury severity	[36]

Amorini AM (2017)	mTBI, weight-drop 450 g from 1-m height; severe TBI, weight-drop 450 g from 2-m height	60 male Wistar rats, weighing 300–350 g, ages not stated	Drop-weight	mTBI caused modest transient changes in NAA, Asp, GABA, Gly and Arg; severe TBI showed greater, long-lasting changes in Glu, Gln, NAA, Asp, GABA, Ser, Gly, Ala, Citr, Tau, Met, SAH, L-Cystat, Tyr and Phe; severity of injury is a major determinant of processes involving amino acid metabolism	[39]

Zander *et al*. (2016)	mTBI and rmTBI, 10–14 psi pressure measured 2 inches above the culture plates	Primary rat cortical neurons from 10 E18 Sprague–Dawley rats, ages not stated	Blast injury	Glutamate release from neurons subjected to three blast injuries was significantly greater than those exposed to single or no injuries	[40]

Raghavendra Rao *et al*. (1998)	TBI type not specified, 4-mm diameter tip at velocity of 3 m/s and 2-mm deformation	Male Sprague–Dawley rats, weighing 250–300 g, ages not stated but reports adult	Injury via controlled cortical impact device	TBI downregulated proteins of glial glutamate transporters GLT-1 and GLAST, which are responsible for clearance of glutamate	[42]

Sullivan *et al*. (1999)	TBI type not specified, 5-mm diameter tip at velocity of 3.5 m/s and to 2-mm depth	70 male Sprague–Dawley rats, weighing 250–300 g, ages not stated but reports young adult	Injury via pneumatically controlled impacting device	Postinjury administration of mitochondrial PTP inhibitor, cyclosporin A, attenuates the disruption of the mitochondrial membrane potential and calcium homeostasis	[44]

Ashwal *et al*. (2004)	TBI type not specified, history of TBI and presumed diffuse axonal injury	38 children and adolescents, age range unspecified	Injury type not specified	Increased glutamate/glutamine levels and lower NAA/Cho in children and adolescents with TBI compared with controls	[46]

Yeo e*t al*. (2011)	mTBI, based on ACRM criteria	30 human subjects, age 27.3 ± 9.52 years	Various (MVA, fall, assault, collision, sports injury, falling object)	Increased glutamate/glutamine levels in the white matter in mTBI human adults when measured at an average of 13 days postinjury	[47]

Okonkwo *et al*. (1999)	TBI type not specified, force of impact device not specified	22 male Sprague–Dawley rats, weighing 375–400 g, ages not stated	Impact acceleration injury	Rats pretreated with cyclosporin A before TBI showed decreased axonal damage and exhibited mitochondrial protection compared with controls	[50]

Buki *et al*. (2000)	TBI type not specified, drop-weight 450 g from 2-m height	25 Sprague–Dawley rats, weighing 365–398 g, ages not stated	Drop-weight	Single TBI resulted in significant increase in cytochrome C release and caspase-3 activation in injured axons; sites of these changes were associated with mitochondrial swelling	[52]

Tweedie *et al*. (2007)	mTBI, drop-weight 30 or 50 g weight from 80-cm height	Male ICR mice, weighing 30–40 g, ages not stated	Drop-weight	mTBI resulted in decreased levels of procaspase-3 and increased levels of Bax	[53]

Abdul-Muneer (2013)	mTBI and rmTBI, 123 kPa intensity blast on blast-wave simulation device	12 male Sprague–Dawley rats, age 11 weeks	Blast injury	rmTBI showed greater activation of caspases compared with single mTBI; mTBI and rmTBI resulted in increased levels of oxidative damage markers in the brain microvessels, increased ROS levels in the brain, and decreased expression of BBB tight-junction proteins	[54]

Huh *et al*. (2007)	mTBI and rmTBI, controlled cortical impact device 5mm diameter tip at velocity of 5 m/s	48 male and female rat subjects, age 11 days	Controlled cortical impact injury	Calpain activation was seen in axons in subcortical white matter tracts of rats subjected to one, two or three mTBIs at day 1 and increased with the number of injuries	[57]

Arrington *et al*. (2006)	Overexpression of Calpain 10, not injury	Kidney mitochondria isolated from male Sprague–Dawley rats (weighing 250 g) and female New Zealand White rabbits (weighing 2 kg)	Overexpression of Calpain 10, not injury	Overexpression of Calpain 10 resulted in morphologic signs of mitochondrial dysfunction; Calpain 10 targeted parts of the electron transport chain in the mitochondria essential for aerobic cellular respiration	[60]

Raghupathi *et al*. (2004)	mTBI and rmTBI, head rotational acceleration in the axial plane using the HYGE pneumatic actuator	11 neonatal farm piglets, ages 3–5 days	Rotational acceleration injury	More regions of axonal damage were seen in pigs that sustained two mild rotational head injuries (10–15 min apart) compared with pigs with single injury	[62]

Laurer *et al*. (2001)	mTBI and rmTBI, controlled cortical impact devide 6-mm diameter tip at velocity 4.8–5.6 m/s	92 male C57BL/6 mice, ages 8–10 weeks	Controlled cortical impact injury	β-APP immunoreactivity (an indicator of traumatic axonal injury) in the brain of rmTBI mice was markedly elevated compared with single mTBI mice	[63]

Shitaka *et al*. (2011)	mTBI and rmTBI, electromagnetic stereotaxic impact device with 9-mm diameter tip at velocity 5 m/s	147 male C57BL/6J wild-type mice, ages 2–3 months	Electromagnetic stereotaxic impact injury	Greater APP immunoreactivity in rats exposed to single and two mTBIs	[64]

Yang MS *et al*. (1985)	mTBI, fluid percussion pulse 1.9–2.2 atm	12 male and female cats, weighing 3–4 kg, ages not stated	Fluid-percussion injury	Brain tissue subjected to single mTBI resulted in increases in markers of impaired brain/mitochondrial metabolism without evidence of cerebral ischemia	[65]

Xing *et al*. (2013)	mTBI, fluid percussion pulse 2.5 atm	38 male Sprague–Dawley rats, weighing 175–275 g, ages not stated but reports adult	Fluid-percussion injury	Brain tissues subjected to single mTBI exhibited reduced expression of mitochondrial proteins involved in the electron transport chain and pyruvate dehydrogenase	[66]

Uryu *et al*. (2002)	mTBI and rmTBI, controlled cortical impact with 6mm diameter tip at velocity 4.8–5.6 m/s	Tg APP695swe and WT mice, age 9 months	Controlled cortical impact injury	rmTBI, but not single mTBI, increase in Abeta deposition and levels as well as levels of isoprostanes, markers of lipid peroxidation	[68]

Amorini *et al*. (2016)	mTBI and severe TBI, drop-weight 450 g from 1 m (mTBI) and 2 m (severe TBI)	90 male Wistar rats, weighing 300–350 g, ages not stated	Drop-weight	mTBI caused late increase in glycolytic gene expression and enzymatic activities; severe TBI resulted in early increase in glycolytic gene expression and enzymatic activities	[67]

Slemmer *et al*. (2002)	mTBI and rmTBI, stretch injury using 94A Cell Injury Controller with 5.5-mm stretch	Primary hippocampal cultures prepared from E18 FVB/N mouse embryos, cells used for experiments within 9–13 days *in vitro*	Stretch injury	rmTBI and mTBI resulted in elevated levels of S-100 beta and NSE (markers of CNS damage) compared with controls; rmTBI caused greater increase in NSE compared with mTBI 6 h postinjury	[69]

Slemmer *et al*. (2004)	mTBI and moderate TBI, stretch injury using 94A Cell Injury Controller with 5.5 mm (mild) and 6.5 mm (moderate) stretch	Primary cerebellar cultures prepared from E18 wild-type FVB/N mouse embryos	Stretch injury	mTBI and moderate TBI resulted in elevated levels of S-100 beta and NSE compared with controls; moderate TBI caused a greater increase in levels of S-100 beta compared with mTBI 24 h postinjury	[70]

Readnower *et al*. (2010)	Moderate blast-induced TBI, blast exposure with peak pressure of 120 kPa	44 male Sprague–Dawley rats, weighing 250–300 g, ages not stated but reports adult	Blast injury	Single blast-induced injury exhibited BBB breakdown in the cortex at three and 24 h postinjury as measured by IgG antibody staining compared with control rats	[72]

Hicks *et al*. (1993)	very mild TBI (0.5 atm), mTBI (1 atm) and moderate TBI (2.2 atm) via fluid percussion injury device	11 male Sprague–Dawley rats, weighing 330–400 g, ages not stated	Fluid-percussion injury	mTBI resulted in BBB disruption as shown by presence of IgG immunoreactivity	[73]

Perez-Polo *et al*. (2013)	mTBI, fluid percussion pulse 1 atm	male Sprague–Dawley rats, weighing 350–400 g, ages not stated	Fluid-percussion injury	Single mTBI resulted in a significant disruption of BBB integrity, reflected by increased IgG antibody and albumin in the brain parenchyma, compared with the control group	[74]

Marchi *et al*. (2013)	Subconcussive head hits	57 human subjects athletes, ages not stated but reports college atheletes	Football-related injuries, no players experienced concussion	Subconcussive injuries showed increased levels of S100B, indicating BBB disruption; elevations in S100B correlated with the number and intensity of the injuries	[75]

Deford *et al*. (2002)	mTBI and rmTBI, drop-weight 50, 100 or 150 g from 40-cm height	117 B6C3F1 male mice, age 9 weeks	Drop-weight	mTBI or rmTBI resulted in no evidence of BBB breakdown; no detection of axonal injury as measured by neurofilament 68 and APP molecules	[76]

ACRM: American Congress of Rehabilitation Medicine; APP: Amyloid precursor protein; BBB: Blood–brain barrier; GCS: Glasgow Coma Scale; mTBI: Mild traumatic brain injury; NAA: N-acetyl aspartate; PTP: Permeability transition pore; rmTBI: Repetitive mTBI.

Of note, the definition of the term concussion is in discussion with regard to the clarification of the terminology. It is difficult to distinguish concussion from mTBI as there is no clear distinction in pathological findings, and inciting injuries are biomechanically similar [19]. In addition, concussion and mTBI share many clinical symptoms including headache, cognitive impairment and sleep disturbance. A review even suggests incorporating concussion into a type of mTBI [19]. The 4th International Conference on Concussion acknowledged concussion as a subset of TBI but did not delineate the difference between concussion and mTBI [20]. In fact, the stated definition of concussion seems to greatly overlap with that of mTBI. Therefore, for the purpose of this review and given the controversy distinguishing the two terms, concussion and mTBI will be determined to be injuries of comparable severity.

## Mitochondria & mitochondrial dysfunction following mTBI & rmTBI

Mitochondria are membrane-bound organelles responsible for energy production via cellular respiration, which is 15-times more efficient at generating the body’s store of energy in the form of adenosine triphosphate (ATP) compared with nonmitochondrial dependent glycolysis alone [21]. The brain consumes 20% of total body oxygen for cellular respiration despite comprising only 2% of the body’s weight; therefore, even slight deprivation of energy from dysfunctional mitochondria may impair neuronal function [22]. While its function of ATP production is important, mitochondria are also involved with other cellular processes. Mitochondrial dysfunction is associated with abnormalities in cellular function and reflects the complexity of this organelle’s involvement in these processes.

Growing evidence points to mitochondrial dysfunction following rmTBI [12,23–25]. The mechanism of mitochondrial dysfunction in rmTBI may occur via opening of the mitochondrial permeability transition pore (PTP) and disruption of the mitochondrial membrane potential. Further, mitochondrial dysfunction has downstream effects leading to injury to the nervous system by activation of caspases, enzymes involved in programmed cell death and calpains, a particular group of calcium-activated proteases. Activation of these enzymes induces many downstream effects leading to negative neurological consequences including neuronal cell death, axonal injury and BBB compromise. We next review the evidence linking mitochondrial dysfunction to mTBI and rmTBI.

## Energetic markers & evidence of mitochondrial impairment in mTBI & rmTBI

The functional state of the mitochondria following rmTBI has been indirectly assessed through the measurement of energetic biomarkers. Many of these studies used technologies such as magnetic resonance spectroscopy (MRS) that noninvasively quantify *in vivo* levels of biomarkers that reflect mitochondrial function. In particular, H-MRS and Phosphorus-MRS are widely employed to quantify levels of brain energetic biomarkers such as *N*-acetyl aspartate (NAA), ATP, lactate and nicotinamide adenine dinucleotide. The NAA molecule is synthesized from aspartate and acetyl CoA in the mitochondria [26], and represents the most visible peak in MRS scans of healthy human brains [27]. An increasing number of studies show that decreases in NAA levels may in fact be a better reflection of impaired mitochondrial function than neuronal death [26,28–30]. NAA is also suggested to be involved in neuronal mitochondrial energy production and helps the neurons to meet its high demand for ATP [31].

The disruption in NAA is seen in single mTBI. Govindaraju and colleagues observed decreases in NAA/Cr and NAA/Cho, markers of neuronal damage or disruption, in 14 human subjects with mTBI compared with 13 control subjects when measured within 1 month postinjury [32]. In addition, Son and colleagues reported a reduction in NAA/Cr ratios in pericontusional regions in seven mTBI human subjects compared with 25 control subjects [33].

Disruption in energetic biomarkers is also evident in rmTBI. In a rodent study, decreased levels of ATP and other energetic markers was observed 2 days after rats were subjected to two mTBIs [23]. ATP, NAA and acetyl-CoA levels were maximally decreased when the interinjury interval was 3 days, even after a 7-day recovery period post second injury. By contrast, rats subjected to an interinjury interval of 5 days did not show differential levels compared with the control group. These results suggest a temporal window between the rmTBIs during which the brain is especially vulnerable and may lend itself to damage that is difficult to reverse.

In a follow-up proton MRS study conducted in human athletes, Vagnozzi and colleagues reported findings that support the presence of mitochondrial impairment following rmTBI [12]. Although this experiment was not originally designed to be an rmTBI study, 3 out of the 13 enrolled athletes endured a second concussion during the course of the study. The NAA/Cr ratio in singly concussed athletes was lower compared with control subjects at 3 days postinjury and recovered to control values at 30 days postinjury. Athletes who sustained a second concussive head injury had identical NAA/Cr ratio to that of athletes with a single concussion when measured at 3 days after the initial injury. However, the second injury (which occurred at 10, 12 and 13 days from the first one) was associated with a further decrease in NAA/Cr ratio post second injury with full restoration taking place at 45 days post initial injury. Thus, having two injuries within a certain window of time – which in this study was an interinjury period of at least 13 days – was associated with longer recovery of these energetic biomarkers. In addition, while singly concussed patients reported resolution of clinical symptoms (including physical, cognitive, emotional and sleep disturbances) within 3 days, all doubly concussed patients symptoms’ persisted up to 30 days. Interestingly, the NAA/Cr ratio did not recover at 30 days in the doubly concussed group despite resolution of clinical symptoms. Thus, patients with decreased NAA levels, signifying impaired brain metabolism, could have greater brain vulnerability to repeat concussion despite absence of clinical symptoms. These results suggest that brain vulnerability is still present in individuals with repetitive concussion despite the lack of detectable clinical symptoms, complicating the decision to ‘clear’ a patient to return to normal activities based on their report of recovery.

A later study from the same research group involving six doubly concussed nonprofessional male athletes with interinjury interval ranging between 9 and 21 days also demonstrated that the repeat concussion increased the time of recovery of NAA/Cr ratio to baseline as well as the persistence of clinical symptoms [34]. These results highlight the complexity of being able to appropriately and accurately determine full rmTBI recovery due to the discrepant clinical and biological data [12].

In addition to NAA, cerebral metabolic rate of glucose (CMRgluc), calculated through spectroscopy, can also provide evidence for brain vulnerability to repeat injury. Decreased CMRgluc levels suggest metabolic dysfunction, as glucose is a substrate used by the mitochondrial and nonmitochondrial methods of ATP production. Rats subjected to a single mTBI show decreased CMRgluc in the parietal cortex and the hippocampus [24]. CMRgluc levels decrease further when rats sustain a second injury within 24 h of the first injury, and remain decreased 3 days postinjury whereas rats subjected to a single mTBI returned to normal CMRgluc values. In contrast, rats that were subjected to 120-h interval rmTBI showed no significant changes compared with that in the group with single mTBI. The CMRgluc in this rmTBI group returned to the levels seen in the uninjured group 3 days after the second injury, suggesting no changes in the CMRgluc recovery time compared with the group with single mTBI. Overall, the lack of significant changes in CMRgluc drop and recovery time between single mTBI group and a longer interinjury interval (120 h) rmTBI group provide evidence that longer interinjury time interval results in less deleterious effects on the injured animal.

Decreased CMRgluc has also been observed in human rmTBI studies. Twelve war veterans with blast-induced rmTBI and persistent postconcussive symptoms exhibited decreased CMRgluc compared with control subjects with no history of brain injury [25]. The decrease in CMRgluc appears to be long lasting as the average time between the veterans’ last blast exposure and the time at which CMRgluc was measured was 3.5 years.

As the evidence reviewed above suggests, there is growing evidence of mitochondrial dysfunction and brain vulnerability in rmTBI in animals and humans. Next, we review the mechanisms by which TBI may lead to mitochondrial dysfunction.

## Mechanism of mitochondrial impairment through the opening of mitochondrial PTP

**Figure 1.**
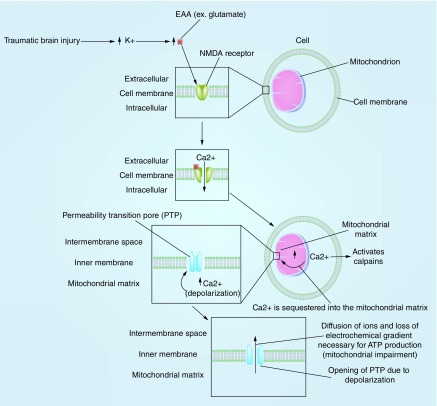
**The process of mitochondrial impairment secondary to disruption of Ca2^+^ homeostasis and opening of permeability transition pore.** PTP: Permeability transition pore.

Mitochondrial dysfunction following TBI starts with an impact to the head. Acceleration–deceleration and rotational forces lead to deformation of neural tissue following TBI and trigger an outflow of potassium from the intracellular to extracellular space, with greater release following more severe injury [35] (Figure 1). Next, the elevation in extracellular potassium ions elicits a release of excitatory amino acids, notably glutamate, by the neurons at the site of the TBI; glutamate levels are also higher for more severe injury compared with mild TBI [36–39]. An *in vitro* study investigating the effect of rmTBI on cultured primary neurons found that glutamate release from neurons subjected to three injuries was significantly greater than those exposed to single or no injuries [40]. As a consequence of increased glutamate levels, NMDA receptors are overstimulated which, in turn, causes an increase in calcium levels [41]. TBI not only leads to increased mitochondrial Ca2^+^ and glutamate levels, but also decreases the clearance of glutamate from the body by downregulating glial glutamate transporters GLT-1 and GLAST [42].

Overstimulation of NMDA receptors and disruption in Ca2^+^ levels contribute to mitochondrial membrane depolarization [43]. The resulting influx of excessive Ca2^+^ into the mitochondria instigates the opening of the mitochondrial PTP and disrupts the mitochondrial membrane potential via further Ca2^+^ influx leading to further depolarization. This ultimately leads to mitochondrial impairment by disrupting the electrochemical gradient necessary for the production of ATP. In fact, postinjury administration of mitochondrial PTP inhibitor, cyclosporin A, attenuates the disruption of the mitochondrial membrane potential and calcium homeostasis [44]. In sum, glutamate-induced NMDA overstimulation results in the disruption of Ca2^+^ homeostasis in the mitochondria and the opening of the PTP, all of which contribute to mitochondrial impairment by disrupting the mitochondrial membrane potential (Figure 1).

Increased glutamate levels, the opening of PTP and the resulting disruption of Ca2+ homeostasis also affect the neuron’s energy demand via depleting ATP stores. The ionic disruption activates ATP-dependent calcium pumps in an effort to restore homeostasis [43]. However, the impaired mitochondria are not able to produce the ATP needed to rectify the ionic disruption. Therefore, this process ultimately results in an increased demand of ATP within the context of lower supply.

Studies show that glutamate levels are indeed elevated in not only animals [36,45] but also humans with TBI. Ashwal and colleagues observed an increase in glutamate/glutamine levels in children and adolescents with TBI [46]. Further, increased glutamate/glutamine levels were observed in the white matter in mTBI human adults when measured at an average of 13 days postinjury [47].

## Mechanism of apoptosis & axonal injury through activation of caspases in the mitochondria

**Figure 2.**
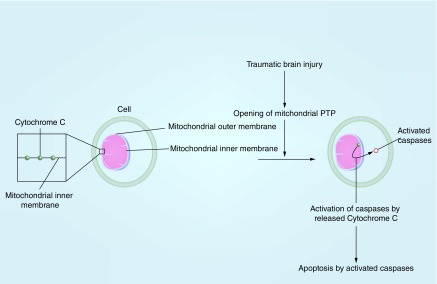
**The mechanism of apoptosis secondary to release of cytochrome C from the mitochondria and the resulting activation of caspases.**

Apoptosis and axonal injury are established neurological consequences of TBI. In addition to reducing production of ATP needed for axonal functions, mitochondrial dysfunction activates caspases that trigger the death of neurons and cleaves structural proteins that maintain the axonal membrane [48]. Mitochondria play an integral role in apoptosis of neurons. The disruption of the mitochondrial transmembrane potential and the opening of PTP are important precipitating factors for apoptosis [49]. Cyclosporin A, a drug that blocks the mitochondrial PTP, was shown to help preserve the mitochondrial membrane potential and delay cell death [49,50]. The mitochondria also contain various proapoptotic proteins, such as cytochrome C. Cytochrome C is embedded in the inner mitochondrial membrane as part of the electron transport chain and mediates the release of itself and other proapoptotic factors during apoptosis [51]. TBI-associated mitochondrial impairment leads to the release of these proapoptotic proteins including cytochrome C (Figure 2). Proapoptotic proteins then trigger the activation of caspases, which are involved with apoptosis and axonal injury [52]. A study showed decreased levels of procaspase-3 and increased Bax, a proapoptotic protein, in mice subjected to mTBI. Procaspase-3 is a proapoptotic protein and the precursor of caspase; thus a decrease in procaspase-3 suggests increased conversion of the precursors to active caspases post mTBI [53]. In addition, an animal study by Abdul-Muneer and colleagues found that rats exposed to repeated mTBI showed increased activation of caspases compared with those subjected to single mTBI [54].

Caspase activation has detrimental consequences to axons. In a study by Buki and colleagues, a single traumatic head injury in rats resulted in the release of cytochrome C and activation of caspases within 15 min of the injury [52]. Axonal segments containing markers for cytochrome C and caspase activation revealed damages that are characteristic of a traumatic axonal injury such as focal cytoskeletal changes.

## Mechanism of axonal injury through activation of calpains

Calpains play an important role in the pathogenesis of traumatic axonal injury in animals and humans. Calpains are calcium-activated cysteine proteases that break down proteins in cells. Thus, these proteases will increase in activity with the increase in calcium concentration associated with rmTBI and loss of calcium homeostasis due to mitochondrial dysfunction [55,56] (Figure 1). In an rmTBI study by Huh and colleagues, calpain activation was seen in axons in subcortical white matter tracts of all injured rats (one, two or three successive injuries) at day 1 and increased with the number of injuries [57]. An animal study that investigated axonal injury in rats after TBI suggests that calpains are involved in axonal injury by damaging axonal cytoskeleton and activating downstream phosphatases [52]. Another study reports that administering calpain inhibitors to rats 10 min after a moderate TBI reduced the losses of cytoskeletal elements of neurons, neurofilament 200 and 68 [58]. Furthermore, an mTBI study showed that calpain-2 inhibitor prevented the degradation of MAP-2, a protein that plays a role in maintaining the cellular structure, in neurons [59].

Calpains are linked to mitochondrial dysfunction. In fact, certain calpains, such as Calpain 10, have been found in the mitochondria. In a study of rat mitochondria, Arrington and colleagues have found that an overexpression of this mitochondrial calpain resulted in morphological signs of mitochondrial dysfunction (i.e., fragmentation and swelling of the mitochondria) [60]. This study also found that Calpain 10 targeted parts of the electron transport chain in the mitochondria that are essential for aerobic cellular respiration. In addition, Calpain 10 may also be linked to mitochondrial permeability transition (PT) as cyclosporin A treatment in these rats preserved normal mitochondrial morphology. In addition, an *in vitro* study of neurons showed that inhibition of calpain by calpastatin protected mitochondria and neurons from the NMDA-mediated excitotoxicity [56]. Thus, calpains may also be involved in the mitochondrial dysfunction seen in patients with rmTBIs.

## Axonal injury in mTBI & rmTBI

Axonal injuries have been reported in rmTBI in animal studies [61] and these studies show a relationship between the mitochondria and such axonal injuries. As previously mentioned, mitochondrial dysfunction from mTBI results in axonal injury via the downstream effects of activating caspases and calpains; in fact, the activation of caspases and calpains is greater in rmTBI compared with single mTBI. As expected, axonal injury after rmTBI is worse compared with one after a single mTBI. For instance, more regions of axonal damage were present in pigs that sustained two mild rotational head injuries (10–15 min apart) compared with pigs with single injury [62]. Further, beta-amyloid precursor protein (APP) immunoreactivity (an indicator of traumatic axonal injury) in the brain of rmTBI mice was markedly elevated compared with single mTBI animals [63] with another study showing greater APP immunoreactivity in rats exposed to single and two mTBIs [64]. These results suggest that rmTBI results in worsened axonal damage compared with a single injury.

Evidence suggests that even a single mTBI leads to mitochondrial damage. For example, a single instance of mTBI has been shown to disrupt energetic biomarkers in both animal models [65] and humans [32,33]. Mitochondrial impairment after a single mTBI is also manifested through decreased expression of mitochondrial proteins. Brain tissues of rats subjected to single mTBI exhibited reduced expression of mitochondrial proteins involved in the electron transport chain and pyruvate dehydrogenase [66]. In an animal study, Amorini and colleagues found that rats exposed to single mTBI showed a transient decrease in ATP levels and three- to fivefold increase in the gene expression of enzymes involved in glycolysis, a significantly less efficient method of producing ATP that does not utilize the mitochondria [67]. These findings suggest that a single mTBI is sufficient to elicit mitochondrial impairment.

The detrimental effects seen in a single mTBI are heightened when the injury becomes repetitive. For example, rmTBI causes greater degree of neuronal damage compared with single mTBI [68,69]. An *in vitro* study by Slemmer and Weber found greater evidence of neuronal damage in hippocampal neurons after repeated mild stretch injuries than after a single mild stretch injury [70]. In fact, the study found that a single injury at subthreshold level of injury, which does not usually cause a noticeable cellular damage, resulted in cellular damage when it was repeated five- to six-times with a 2-min interinjury period. Thus, with evidence of greater neuronal and axonal damage associated with rmTBI and the link between mitochondrial impairment and these events, it would be reasonable to expect a greater severity of mitochondrial impairment in rmTBI. In fact, there is increasing evidence demonstrating that rmTBI has greater negative consequences for mitochondrial function than single mTBI.

Several studies have utilized cyclosporin A, a substance that inhibits the opening of mitochondrial PTP, in order to demonstrate the link between mitochondrial impairment and axonal damage. Rats given cyclosporin A 30 min before sustaining a single TBI exhibited reduced APP immunoreactivity, suggesting decreased axonal damage, when compared with the rats that were not pretreated with cyclosporin A [50]. Buki and colleagues also found reduced axonal damage in rats when they were administered cyclosporin A 30 min after a single TBI [52].

## rmTBI & blood–brain barrier

Thus far, our review discussed the mechanisms of mitochondrial impairment post-TBI, the downstream activation of caspases and calpains as well as their harmful effects on neurons and axons. While these mechanisms have been studied more extensively in moderate-to-severe head injuries, studies show that mitochondrial impairment and axonal injury are also indicated in mTBI. The mechanism involving increased glutamate levels, opening of mitochondrial PTP and the downstream disruption of the mitochondrial transmembrane potential are suggested in mTBI. Emerging evidence suggests altered biomarker levels and poorer clinical outcomes associated with rmTBI, especially with shorter interinjury interval, compared with single mTBI. In the next section, the review investigates the effects of mitochondrial dysfunction on the BBB, an important structure that protects the brain.

The mechanism of mitochondrial dysfunction may also be related to disruption of the BBB post mTBI. The BBB appropriately shields the brain from many substances in the blood; however, the integrity of the BBB may be compromised in pathological conditions, including Alzheimer’s disease, brain tumors and multiple sclerosis [71]. BBB breakdown can lead to a number of negative outcomes, such as dysregulation of ions in brain interstitial fluid for optimal neuronal function and exposure of the brain to peripherally circulating agents [71]. One method of determining the integrity of BBB is by measuring Immunoglobulin G (IgG) antibody, which cannot cross an intact BBB. Thus, the presence of IgG antibody in the brain parenchyma would reflect BBB breakdown.

Several studies indicate the presence of BBB compromise after a TBI. Rats exposed to a single blast-induced injury exhibited BBB breakdown in the cortex at 3 and 24 h postinjury as measured by IgG antibody staining compared with control rats [72]. To note, BBB breakdown was also seen in other forms of TBI (in addition to blast-induced TBI injury). Hicks and colleagues also confirmed the presence of BBB breakdown in rats with mTBI as measured by IgG immunocytochemistry [73]. An animal study by Perez-Polo and colleagues found that rats subjected to single mTBI showed a significant disruption of BBB integrity, reflected by increased IgG antibody and albumin in the brain parenchyma, compared with the control group [74]. Evidence shows that BBB breakdown may be worse with rmTBI compared with mTBI. Three out of five rodents that sustained a single mTBI exhibited a small area of IgG staining at 24 and 48 h postinjury, suggesting some BBB breakdown [63]. Not surprisingly, four out of five rodents that sustained rmTBI exhibited a more noticeable and widespread area of IgG staining, suggesting a more significant compromise of the BBB with repeated injuries.

Although human subjects have been less studied, there is some evidence of BBB breakdown in humans after mTBI. Recent findings show evidence for BBB disruption in athletes with repeated subconcussive head injuries [75]. BBB disruption as measured by serum levels of S100β, a marker for BBB breakdown, was seen in football players who sustained subconcussive head injuries that were not severe enough to be qualified for a diagnosis of concussion. In support of the notion that repeated head injuries lead to a more negative prognosis compared with a single injury, changes in S100β levels were positively correlated with the frequency and the severity of the head injuries.

Despite findings demonstrating the presence of BBB compromise in mTBI, other studies show that mTBI and even rmTBI do not provide a sufficient impact to cause the breakdown of the BBB. In a study by DeFord and colleagues, mice (n = 117) that sustained head injuries using a weight-drop model (weights ranged from 50 to 150 grams dropped from 0.4-m height) were measured for IgG detection [76]. Neither single mTBI nor rmTBI resulted in BBB breakdown. Additionally, there was no detection of axonal injury as measured by neurofilament 68 and APP molecules. On the other hand, the mice that sustained rmTBI using the 100 and 150 grams showed a significantly decreased cognitive function (measured via Morris water maze assessment) compared with noninjured and singly injured mice. Another study using a weight-drop model defined mild brain injury under the conditions of using heavier weights that were dropped at higher heights (450 g weights dropped from 1 m height) compared with DeFord’s study [77]. This study reported diffuse axonal injury as well as posttraumatic brain edema, a sign of BBB dysfunction, in the rats exposed to mild brain injury. It is currently unclear whether the discrepant results are due to differences in drop weight, procedures, or other methodological differences. Additional research is necessary to refine animal models of TBI and the severity level necessary to initiate BBB compromise.

## Mitochondrial dysfunction, caspases, reactive oxygen species & BBB

**Figure 3.**
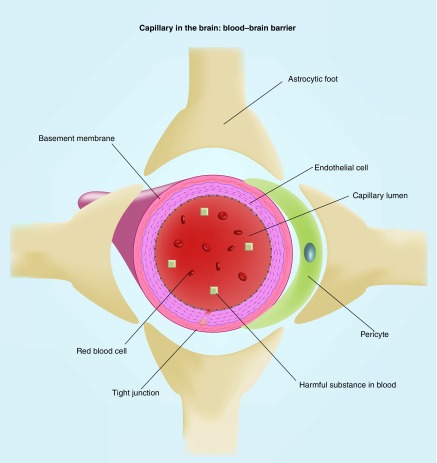
**A schematic of blood–brain barrier and capillary in the brain.** Structures boxed in blue are components of the BBB. BBB: Blood–brain barrier.

As discussed previously, evidence suggests that head trauma leads to the activation of caspases via cytochrome C, which can lead to apoptosis and axonal injury. The BBB is comprised of endothelial cells, basement membrane, tight junctions, astrocytic feet and pericytes, which are all involved with keeping the BBB intact [78] (Figure 3). Increased caspase activation (via increased cytokine concentration) is also associated with increased BBB breakdown and increased cell apoptosis [79]. This may suggest that increased caspase activation in mTBI may result in further apoptosis of the elements that are essential in maintaining the integrity of the BBB.

Further, it was described earlier that rmTBI may lead to mitochondrial PT via the disruption of the mitochondrial transmembrane potential and opening of the mitochondrial PTP; interestingly, this mechanism may also be involved with BBB breakdown through the production of reactive oxygen species (ROS), a group of free radicals. Opening of the mitochondrial PTP via calcium is suggested to be accompanied by increased mitochondrial production of ROS, suggesting that PT may augment mitochondrial oxidative stress [80]. Further, preventing opening of the mitochondrial PTP via the administration of cyclosporin A 15 min postinjury reduced the formation of reactive oxygen species [43].

**Figure 4.**
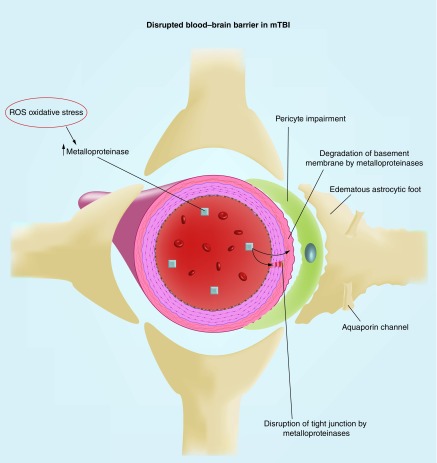
**A schematic of a disrupted blood–brain barrier.** The end results of the BBB impairment are: the disruption of tight junctions that hold together the endothelial cells of the blood vessels, the gradation of basement membrane and the impairment of pericytes and the edematous astrocytic feet that are parts of the perivascular component crucial for the integrity of BBB. BBB: Blood–brain barrier.

An animal study by Abdul-Muneer and colleagues further investigated the role of ROS in increasing BBB permeability in mTBI and rmTBI [53] (Figure 4). Rats exposed to single mTBI (123-kPa shock-wave exposure) and repeated mTBI (with 24-h interinjury interval) showed increased levels of oxidative damage markers in the brain microvessels and increased ROS levels in the brain. Interestingly, rats exposed to repeated mTBI (with 24-h interinjury interval) showed an even greater increase in a marker of ROS generation compared with when they were exposed to single mTBI. These changes were temporally linked with a decreased expression of BBB tight-junction proteins compared with the control group. Further, rats exposed to single mTBI and rmTBI showed a similarly decreased expression of PDGFR-β, a marker of supporting pericytic cells of the BBB. There was also evidence for activation of metalloproteinases, which degrade basement membrane proteins and tight junctions, and the resulting increased expression of aquaporin channels (a water channel protein) around the perivascular region (specifically astrocyte end-feet) in the brain tissue of mTBI and rmTBI rats. The aquaporin channels are suggested to increase permeability of the BBB. In addition, an *in vitro* study of cultured neurons found that those subjected to three mTBIs showed greater release of ROS compared with those exposed to single or no mTBIs [39]. In another study, rats subjected to rmTBI (three injuries 5 days apart) exhibited increased markers of oxidative stress (lipid peroxidation) in the brain tissue [81]. In fact, administration of progesterone, which has an antioxidative effect, resulted in less lipid peroxidation and better cognitive outcomes.

An experimental animal study using cell cultures from rat and human brains supports the role ROS has in disrupting the BBB [82]. This study found that ROS disrupted the integrity of the tight junctions comprising the BBB. Further, the transendothelial electrical resistance was decreased in ROS-injured rat and human brain endothelial cells, indicating a disruption in BBB integrity. ROS also initiated changes to the cytoskeleton and disappearance of proteins critical for maintaining tight junction integrity (occludin and claudin-5).

## Summary & future perspective

We reviewed the mechanism of mitochondrial impairment in TBI, including activation of caspases and calpains, and presented evidence of mitochondrial involvement in TBI through alterations in biomarkers. The neurological consequences of mitochondrial impairment including neuronal apoptosis, axonal injury and BBB disruption were also highlighted. Further, we provided evidence for potentially worse mitochondrial dysfunction associated with rmTBI compared with single mTBI.

There remain several areas that would benefit from further research. First, there is a need to identify additional biomarkers of mitochondrial dysfunction. One promising marker that could be further explored is peripheral blood level of mitochondrial DNA post-TBI as a biomarker for cerebral mitochondrial energetics impairment [83]. Exploration of novel biomarkers is not only important in determining the level of mitochondrial dysfunction but also understanding its pathophysiology. For example, patients with certain genomic variant of mitochondrial DNA, haplogroup K, were reported to have a more favorable outcome in Glasglow Outcome Score post-TBI [84]. Increasing age is a known strong determinant of worse outcomes of TBI. Patients with haplogroup K showed a slower decline in Glasglow Outcome Score with increasing age compared other haplogroups. This suggests that there may be an inherent link between mitochondrial genome and outcome of TBI. It would be meaningful to explore this further in order to determine the role that this particular mitochondrial genomic variant has in the pathophysiology of mitochondrial dysfunction.

Another area of future research is validating animal models of mTBI. There are several different methods of inducing injury in animal studies including blast and drop-weight models. While these models are valuable because they mimic different situations of TBI in the real world, it would be interesting to consider if the methods of injury have an impact on the severity of TBI. In fact, an animal study comparing lateral fluid percussion model and weight-drop with hypoxia model of comparable injury severity found discrepancies in motor recovery periods (the rats under the weight-drop model recovered by 14 days vs 6 weeks for the lateral fluid percussion model), learning and memory deficits (present for lateral fluid percussion model vs absent in weight-drop model) as well as severity of neuronal degeneration (greater degree of neuronal degeneration for lateral fluid percussion model) [85]. Thus, even with similar injury severity, a certain mode of injury may result in a more detrimental effect compared with another mode of injury. Studying these injury models more thoroughly will allow future investigations to help design experiments with more accurate level of injury severity and allow comparisons between studies with different injury methods.

Many rmTBI animal studies discussed in this review subjected the animals to two injuries. As these injuries tend to occur more frequently in certain professions, it would be important to study models that subject the animals to more than two injuries to further investigate the degree of cumulative effects of these repeated injuries. With regard to the critical interinjury interval period, it would be helpful to identify a temporal window during which subsequent injury causes a cumulative harm. Understandably, it is difficult to determine this window using animal models because the temporal window in the animal model may not translate to humans. Careful investigations with recruitment of real-life TBI patients and monitoring need to be done in order to determine this critical temporal window. By determining the critical window, one can better estimate the prognosis of negative consequences associated with repetitive TBIs with a certain interinjury interval.

Evidence also suggests an association between neurodegenerative and psychiatric disorders and TBI [86–88]. For example, increased risks of neurodegenerative disorders such as Alzheimer’s disease and amyotrophic lateral sclerosis are suggested for patients post TBI [86]. Similarly, patients with TBI have increased risks of psychiatric disorders such as depression, bipolar disorders and post-traumatic stress disorder (PTSD) [88,89]. Considering the link between mitochondria and TBI, it would be interesting to investigate whether or not there is an overlap of mechanism between these psychiatric disease processes and TBI.

There has also been emerging research on the different types of therapy for patients post TBI. Examples include hyperbaric oxygen therapy, hypothermia therapy and transcranial light therapy [90–94]. Specifically for rmTBI, Huang and colleagues found that rats subjected to rmTBI (two injuries with a 3-day interinjury interval) treated with daily hyperbaric oxygen therapy during the interinjury period had reduced histopathological gliosis and pathological MRI finding associated with the second injury [90]. Further, rats exposed to rmTBI (two injuries) that were treated with 1-h of hypothermia an hour after the second injury had axons with decreased APP density compared with the untreated group [93]. This study highlights that even a delayed treatment of hypothermia may still have therapeutic effects in rmTBI. Further research in the field of treatment is necessary as there is still debate among the efficacies of different therapies.

**Executive summary**

**Background**

- Mild traumatic brain injury (mTBI) is a significant national health concern and there is growing evidence that repetitive mTBI (rmTBI) can cause long-term change in brain structure and function. The purpose of this review is to synthesize the mechanism of mitochondrial dysfunction in rmTBI and its downstream effects on neuronal cell death, axonal injury and blood–brain barrier (BBB) compromise.

**Findings**

- Evidence suggests that mTBI is severe enough to induce mitochondrial dysfunction. The resulting mitochondrial dysfunction has been shown to trigger a cascade of events including ionic changes and glutamate excitoxicity that lead to activation of enzymes known as caspases and calpains. Activation of these enzymes induces many downstream effects leading to negative neurological consequences including neuronal cell death, axonal injury and BBB compromise. In addition, compared with an isolated mTBI, research suggests that repeated mTBI (rmTBI) may lead to more severe neurological consequences.

**Conclusion & future perspective**

- The findings as a whole demonstrate that the mechanisms of mitochondrial impairment, axonal injury and BBB disruption are interconnected and very complex. In the realm of rmTBI, there may be a critical temporal window of vulnerability between injuries during which damage caused by the subsequent injury is more harmful. Further areas of research include biomarkers, different injury models, long-term effects, association with other diseases and therapy. With practical relevance of rmTBI to the general population and certain professions, this topic remains an important field of further research.
